# A Comparative Analysis of the Chloroplast Genomes of Four *Polygonum* Medicinal Plants

**DOI:** 10.3389/fgene.2022.764534

**Published:** 2022-04-25

**Authors:** Shuai Guo, Xuejiao Liao, Shiyu Chen, Baosheng Liao, Yiming Guo, Ruiyang Cheng, Shuiming Xiao, Haoyu Hu, Jun Chen, Jin Pei, Yangjin Chen, Jiang Xu, Shilin Chen

**Affiliations:** ^1^ Pharmacy College, Chengdu University of Traditional Chinese Medicine, Chengdu, China; ^2^ Institute of Chinese Materia Medica, China Academy of Chinese Medical Sciences, Beijing, China; ^3^ Kenneth P. Dietrich School of Arts and Sciences, University of Pittsburgh, Pittsburgh, PA, United States; ^4^ Beijing Engineering Research Center of Pediatric Surgery, Engineering and Transformation Center, Beijing Children’s Hospital, National Center for Children’s Health, Capital Medical University, Beijing, China; ^5^ Department of City and Regional Planning, Nanjing University, Nanjing, China

**Keywords:** *Polygonum*, comparative analysis, phylogenetic analysis, repeats analysis, complete chloroplast genome

## Abstract

*Polygonum* is a generalized genus of the Polygonaceae family that includes various herbaceous plants. In order to provide aid in understanding the evolutionary and phylogenetic relationship in *Polygonum* at the chloroplast (cp) genome-scale level, we sequenced and annotated the complete chloroplast genomes of four *Polygonum* species using next-generation sequencing technology and CpGAVAS. Then, repeat sequences, IR contractions, and expansion and transformation sites of chloroplast genomes of four *Polygonum* species were studied, and a phylogenetic tree was built using the chloroplast genomes of *Polygonum*. The results indicated that the chloroplast genome construction of *Polygonum* also displayed characteristic four types of results, comparable to the published chloroplast genome of recorded angiosperms. The chloroplast genomes of the four *Polygonum* plants are highly consistent in genome size (159,015 bp–163,461 bp), number of genes (112 genes, including 78 protein-coding genes, 30 tRNA genes, and 4 rRNA genes), gene types, gene order, codon usage, and repeat sequence distribution, which identifies the high preservation among the *Polygonum* chloroplast genomes. The *Polygonum* phylogenetic tree was recreated by a full sequence of the chloroplast genome, which illustrates that the *P. bistorta*, *P. orientale*, and *P. perfoliatum* are divided into the same branch, and *P. aviculare* belongs to *Fallopia*. The precise system site of lots base parts requires further verification, but the study would provide a basis for developing the available genetic resources and evolutionary relationships of *Polygonum*.

## Introduction

*Polygonum* is an annual genus of Polygonaceae, which are broadly spread around the world, and most of them are distributed in the north temperate zone (Macêdo et al., 2021; Mohtashami et al., 2021; Song et al., 2020). Moreover, there are about 113 species in China. Some species of Polygonaceae have been used as traditional Chinese medicine due to their remarkable effects on the treatment of edema and sore poison. *Polygonum aviculare*, *Polygonum bistorta*, *Polygonum cuspidatum*, and *Polygonum perfoliatum* have displayed medical values as diuretics in clinical practice, and it has effects of dehumidification of the wind, heat detoxification, and live blood (Lin et al., 2015). Phytochemical research studies showed that flavonoids, quinones, phenylpropanoids, and terpenoids are contained among *Polygonum*. Also, the pure compounds from *Polygonum* might have extensive bioactivities, such as anticancer, antitumor, antioxidative, anti-inflammatory, analgesic, antimicrobial, and insecticidal activities. The subordinate division of Polygonaceae is not clear and has been controversial. Since Linnaeus established the genus *Polygonum* in 1753, Meissner conducted an in-depth research on the genus *Polygonum* in the world in 1826 and established 10 groups under the genus of *Polygonum* (Fan et al., 2013). With the further development of research work, most groups have been promoted to the genus level by some scholars, and the genus is divided into 15 genera (Costea and Tardif 2005). In the different genera of *Polygonum*, the medicinal chemical components and the curative effects on diseases are different. Therefore, accurate identification of the species is the key to ensuring the clinical efficacy and safety of medicinal plants of this genus.

Chloroplast is one of the plastids and a vital organelle for transforming the energy and performing photosynthesis among the plants, which can be generally found in land plants, algae, and some protists (Asaf, Khan, Aaqil Khan, et al., 2017). Chloroplasts are composed of membranes, with thylakoids and stromata inside. The membrane of the thylakoid contains a large number of pigment molecules in photosynthesis, which are used to capture and transfer energy during photosynthesis, while the stroma contains various enzymes, inorganic salts, and DNA. Chloroplasts can not only synthesize sugars into photosynthesis but also participate in the synthesis of complex organic substances such as amino acids and fatty acids in organisms (Yao et al., 2015). The chloroplast genomes are conversed into many plants, presenting a covalently closed circular DNA, and few are linear or other shapes (Asaf et al., 2017). For example, in *Acetabularia*, the chloroplasts are found to be a rare linear structure rather than a conventional closed circular double-stranded DNA, and in another alga, a polycyclic structure exists because several independent micro-circular linked to each other in dinoflagellate (Cheng et al., 2017; Cheon et al., 2019; Yu, Sun, et al., 2020). The size of chloroplast genomes is usually between 100 and 200 kb in plants of different families and genera of plants (Chen et al., 2019). The chloroplast DNA size of most angiosperms is generally between 110 and 160 kb, and the chloroplast DNA size of ferns is about 140–150 kb.

The double-stranded closed circle of the chloroplast genome is generally classified into four regions: large single-copy region (LSC), small single-copy region (SSC), inverted repeat region A (IRa), and inverted repeat region B (IRb). Two IR regions are separated by LSC and SSC, and they have the same length in opposite directions (Jansen et al., 2011). Studies (Jansen, Saski, Lee, Hansen and Daniell 2011; Cheon, Kim, Kwak, Lee and Yoo 2019) have shown that changes in the IR region are the main reason for chloroplast genome changes. In the earlier reported cp genome in Polygonaceae, Yu and Ye (Yu et al., 2020; Ye et al., 2021) studied the length of the cp genome of LSC, SSC, and IR region and classified the species of *Polygonum chinense* and *P. cuspidatum*. The result would be a valuable genetic resource for studying the genetics and evolutionary relationships between the Polygonaceae species. There are about 110–130 genes encoded by chloroplast DNA, which consist of rRNA-coding genes, protein-coding genes, and tRNA-coding genes (Cheng, Li, Zhang, Cai, Gao, Qiao and Mi 2017; Tan et al., 2020). In general, gene replication occurs in all rRNA genes, along with some protein-coding and tRNA genes. Based on the functions of chloroplast DNA-encoding genes, it can be divided into three classes: genes for the photosynthetic system, such as *petB*, genes for the genetic system related to transcription and translation, such as *tRNA-UGC*, genes for biosynthesis related to the synthesis of amino acids, and open reading frames (ORF), such as *accD*, *matK*, and *ycf1* (Yang et al., 2013).

The chloroplast genome is a valuable multi-level taxonomic resource with rich genetic information and has been broadly used in the aspect of plant phylogeny and evolution, species identification, and taxonomy. One reason for the fast development of chloroplast genomes is the advent of high-throughput sequencing technologies. After the complete chloroplast genomes of *Nicotiana tabacum* (Shinozaki et al., 1986) and *Marchantia polymorpha* (Kohchi et al., 1988) were established in 1986, researchers (Daniell et al., 2016) have paid more attention to chloroplast genomes of plants. After that, 3,721 cp DNA in different plants has been described, including green algae and aquatic life which can be found in National Center for Biotechnology Information (NCBI) database (Tonti-Filippini et al., 2017). First-generation sequencing technologies (Pareek et al., 2011; Slatko et al., 2011; Liao et al., 2021), including the traditional “dideoxy” sequencing technique, chemical degradation method, and the improved fluorescence automatic sequencing technology developed based on them (Dobrogojski et al., 2020). The next-generation sequencing technology that does not require DNA amplification and cloning and the third-generation sequencing technology that uses single-molecule real-time (SMRT) sequencing is now being widely used in chloroplast genome sequencing, which could facilitate *de novo* genome assembly (Tonti-Filippini, Nevill, Dixon and Small 2017).

Most of the *Polygonum* species have high financial and therapeutic values. Here, four *Polygonum* chloroplast genomes (*P. aviculare*, *P. bistorta*, *P. orientale*, *P. perfoliatum*) were identified and assembled, compared with other published *Polygonum* chloroplast genomes (Yu, Liu, Liu, Lan and Qu 2020; Ye, Lin, Zhou, He, Yan and Cheng 2021), we got considerable biological evidence, with the cp genome structure, repeat sequences, and other characteristics. This work also provided an essential foundation of the *Polygonum* cp genome library, encouraging the progress of phylogenetics, DNA barcoding, and population (Lee et al., 2019).

## Materials and Methods

### DNA Extracting, Sequencing, and Genome Annotation

DNA was extracted from four species of *Polygonum*, such as *P. aviculare*, *P. bistorta*, *P. orientale* and *P. perfoliatum*. Fresh *Polygonum* leaves were collected from Chengdu (Sichuan Province, China). The specimens of *Polygonum* have been kept in CDCM (Traditional Chinese medicine herbarium of Chengdu University of Traditional Chinese Medicine) (Supplementary Table S1). The cetyltrimethylammonium bromide method has been used to obtain the whole genome DNA from fresh leaves. The ND-2000 spectrometer was used to quantify the DNA. A shotgun library (250 bp) was built following the constructer guidelines. The X Ten Platform (Illumina, San Diego, CA, United States) was used to sequence through the double terminal sequencing technique with pair-end 150. The total raw data from the mensuration DNA was about 3.5G, and about 12 million paired-ends scrutinizes were finished (Supplementary Table S2).

The raw reads were trimmed using Skewer v0.22 (skewer -q 20 -Q 30–l 100 -t 32) (Jiang et al., 2014). BLAST was used to predict the chloroplast-like reads by cleaning the reads with the sequences of the reference *P. cuspidatum* (MW411186.1) (Deng et al., 2015; Ye, Lin, Zhou, He, Yan and Cheng 2021). Generally, SOAPdenovo-2. 04 (SOAPdenovo-127mer all -s config. txt -o out -K 51 -R) was used to accumulate the sequences using chloroplast reads (Gogniashvili et al., 2015). Then, those accumulated assembled sequences were extended with the help of SSPACE-3. 0 (SSPACE_standard_v3.0.pl -l library.txt -s out.config -x 1 -T 4 -b sspace.out) and GapCloser-1.12 (GapCloser -a scaffols.fa -b library.txt -o Fanal.fa)were used to fill the gaps (Boetzer et al., 2011; Acemel et al., 2016). To authenticate the accuracy of the connection splicing, a random primer was designed to check the connections of the sequence by polymerase chain reaction. The PCR primer information and amplification conditions are shown in the Supplementary Table S3. The Sanger sequencing results were compared with the assembled chloroplast genome sequence to verify the accuracy of genome linkage.

CpGAVAS (Liu et al., 2012) was used for sequence annotation. The annotation results were checked by DOGMA (http://dogma.ccbb.utexas.edu/) and BLAST (Wyman et al., 2004). In addition, the tRNA genes were classified using tRNAscanSEv1. 21 (Chan and Lowe 2019). The OGDRAWv1. 2 (Lohse et al., 2007) and MEGA5. 2 (Tamura et al., 2011) were used to plot the structural features of the chloroplast genome and define the relative utilization of synonymous codons. MEGA5. 2 (Tamura, Peterson, Peterson, Stecher, Nei and Kumar 2011) was adopted to analyze the relative synonymous codon usage (RSCU). The assembled chloroplast genome sequences of the four *Polygonum* species were deposited in NCBI under the Genbank accession number MZ748474–MZ748477.

### Repeats and Comparative Analysis of Chloroplast Genomes

Tandem Repeats Finder (Benson 1999) and REPuter (Kurtz et al., 2001) have been used to find the tandem, forward, and palindromic repeats from the four *Polygonum* chloroplast genomes. The Misa.pl (Beier et al., 2017) was used to recognize the SSRs and the finding parameters of mononucleotides transfer to eight repeatable elements, dinucleotides, and trinucleotides four repeatable elements, tetranucleotides, pentanucleotides, and hexanucleotides transfer to three repeatable elements. Primer3 (Untergasser et al., 2012) was used to design the SSR primers.

Genome structures among six *Polygonum* chloroplast genomes, Including four *polygonum* species in this study and two *polygonum* species published by the NCBI (*P. cuspidatum* and *P. chinense*), were completed by mVISTA software (Shuffle-LAGAN mode) (Frazer et al., 2004) using the genome of *P. cuspidatum* as the reference. Pi values and sequence polymorphisms of six *Polygonum* species were analyzed using DNAsp v. 6.12.03 (Rozas et al., 2017). The step size was set to 200 bp, and the window length was set to 800 bp.

### Phylogenetic Analysis

A total of 19 chloroplasts sequences (Supplementary Table S4) were used to build the phylogenetic trees. Each of the 67 protein-coding genes shared by all the genomes was compared individually and then linked end to end to form a supergene from each species. The sequences alignment was carried out using the MAFFT v7.309. The best model was determined using the modeltest-ng-0.1.6 software with default parameters; ML analysis was performed using RAxMLNG v0.9.084 based on Linux edition using default parameters. The parameters were GTR + FU + IU + G4m, noname = 1–51,039. *Chrysanthemum x morifolium* has been situated likewise those out-groups.

## Results

### Features of *Polygonum* Chloroplast DNA

The genome sizes of the four *Polygonum* chloroplasts are 163,461 bp (*P. aviculare*), 159,476 bp (*P. bistorta*), 159,015 bp (*P. orientale*), and 160,680 bp (*P. perfoliatum*), respectively. The whole GC content are 37.43% (*P. aviculare*), 37.37% (*P. bistorta*), 38.21% (*P. orientale*), and 37.96% (*P. perfoliatum*), individually. The LSC region, SSC region, and a couple of inverted repeat regions (IRA/IRB) are alike in *Polygonum* chloroplast genomes than other plants (Guo et al., 2018). The length of the LSC region is 83,583–88,021 bp and the GC content is 35.48–36.59% in *Polygonum* chloroplast genomes. The distribution of length in the SSC regions is 12,928–13,306 bp and the GC content is about 32.46–33.19%. The GC content in those IR regions is 41.27–41.45% and the length is 31,067–31,184 bp (Table 1). The GC content is a significant marker to identify the genetic relationship of species; moreover, the *Polygonum* has comparable cpDNA GC content. The phenomenon is also common in other plants (Guo, Guo, Zhao, Xu, Li, Zhang, Shen, Wu and Hou 2018; Liang et al., 2019) that the GC content in those IR regions is more than that of other regions (LSC, SSC). The high GC content of the IR areas usually points to the rRNA and tRNA genes (He et al., 2016; Shen et al., 2017). Also, the whole chloroplast genome sequences of four *Polygonum* species can be checked in the National Center for Biotechnology Information (NCBI) database afterward annotation and the GenBank accession number can be found in Table1.

**TABLE 1:** Chloroplast genome features of *Polygonum*.

Species	All		LSC		SSC		IR		Accession numbers
	Length (bp)	GC%	Length (bp)	GC%	Length (bp)	GC%	Length (bp)	GC%	
*Polygonum aviculare*	163,461	37.43	88,021	35.48	13,306	32.46	31,067	41.27	MZ748474
*Polygonum bistorta*	159,476	37.37	84,360	36.07	12,968	32.99	31,074	41.35	MZ748475
*Polygonum orientale*	159,015	38.21	83,583	36.59	13,154	33.19	31,139	41.45	MZ748476
*Polygonum perfoliatum*	160,680	37.96	85,384	36.20	12,928	33.09	31,184	41.39	MZ748477

**FIGURE 1:**
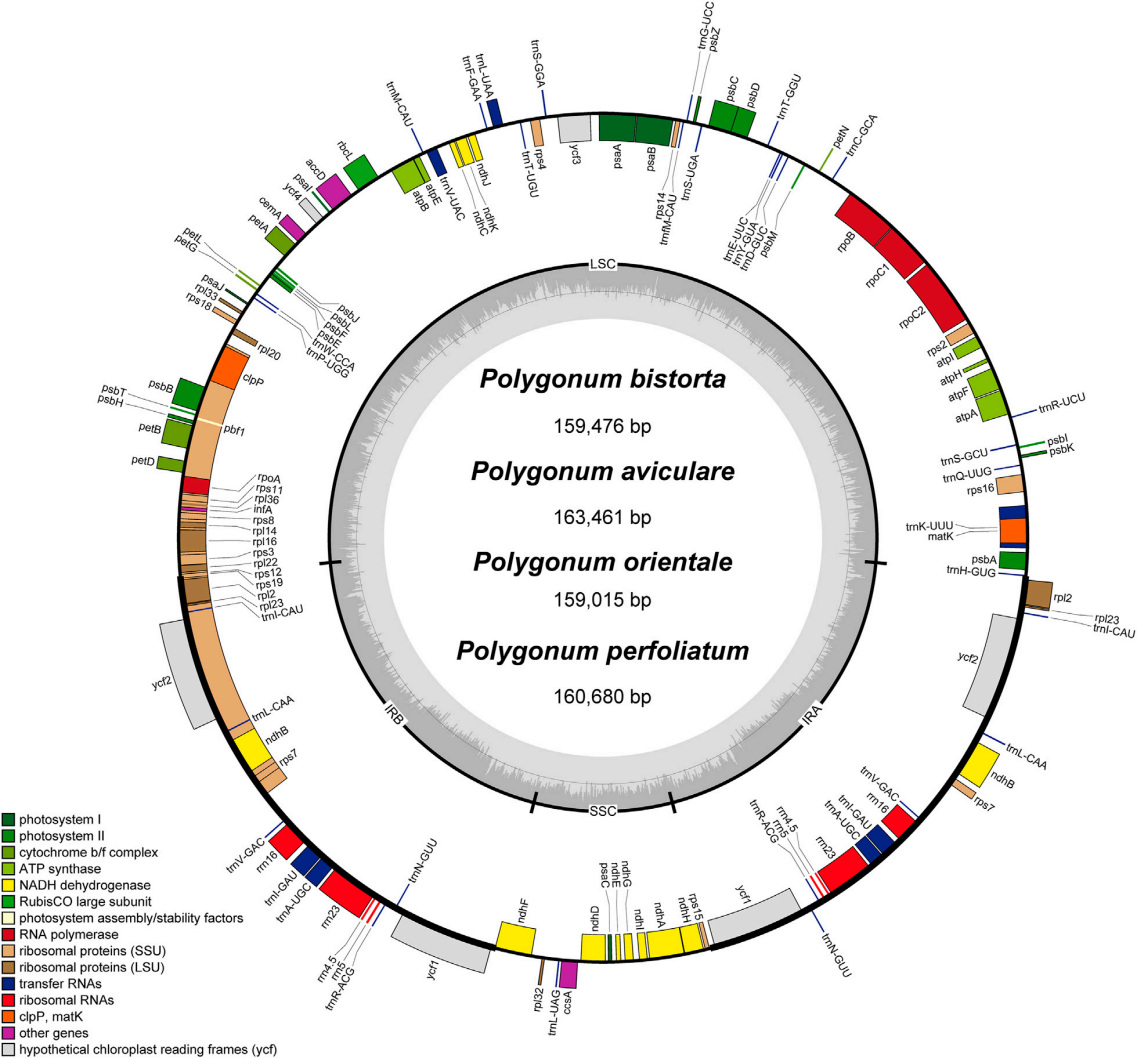
Gene map of the *Polygonum* chloroplast genome. Genes drawn inside the circle are transcribed clockwise, and those outside are transcribed counterclockwise. Genes belonging to different functional groups are color coded. The darker gray in the inner circle corresponds to DNA G + C content, while the lighter gray corresponds to A + T content.

The annotation results (GB files) of four *Polygonum* chloroplast genomes which were measured in this study were submitted to the OGDraw software, and the physical map of the *Polygonum* chloroplast genomes were drawn. The results can be found in Figure 1. In total 112 genes were found in the chloroplast genomes, such as four rRNA genes, 30 tRNA genes, and 78 protein-coding genes (Figure 1; Table 2). The main genes of the four *Polygonum* chloroplasts can be coarsely divided into three classes, named chloroplast self-replication-related genes, photosynthesis-related genes, and other genes (Saski et al., 2005).

**TABLE 2:** Gene composition of the chloroplast genome of *Polygonum*.

Category	Group of genes	Name of genes
Self-replication	Large subunit of ribosomal proteins	*rpl2* ^a^ ^,^ ^b^ *, 14, 16* ^a^ *, 20, 22* ^b^ *, 32, 33, 36*
	Small subunit of ribosomal proteins	*rps2, 3, 4, 7* ^b^ *, 8, 11, 12* ^a^ ^,^ ^b^ *, 14,15, 16* ^a^ *, 18, 19* ^b^
	DNA-dependent RNA polymerase	*rpoA, B, C1* ^a^ *, C2*
	rRNA genes	*rrn16S* ^b^ *, rrn23S* ^b^ *, rrn4.5S* ^b^ *, rrn5S* ^b^
	tRNA genes	*trnA-UGC* ^a^ ^,^ ^b^ *, trnC-GCA, trnD-GUC, trnE-UUC, trnF-GAA, trnfM-CAU, trnG-UCC, trnG-GCC, trnH-GUG, trnI-CAU, trnI-GAU* ^a^ ^,^ ^b^ *, trnK-UUU* ^a^ *, trnL-CAA, trnL-UAA, trnL-UAG, trnM-CAU, trnN-GUU, trnP-UGG, trnQ-UUG, trnR-ACG, trnR-UCU, trnS-GCU, trnS-GGA, trnS-UGA, trnT-GGU, trnT-UGU, trnV-GAC, trnV-UAC* ^a^ *, trnW-CCA, trnY-GUA*
Photosynthesis	Photosystem I	*psaA, B, C, I, J*
	Photosystem II	*psbA, B, C, D, E, F, H, I, J, K, L, M, N, T, Z,*
	NADH oxidoreductase	*ndhA* ^a^ *, B* ^a^ ^,^ ^b^ *, C, D, E, F, G, H, I, J, K*
	Cytochrome b6/f complex	*petA, B* ^a^ *, D* ^a^ *, G, L, N*
	ATP synthase	*atpA, B, E, F* ^a^ *, H, I*
	Rubisco	*rbcL*
Other genes	Maturase	*matK*
	Protease	*clpP* ^a^
	Envelope membrane protein	*cemA*
	Subunit acetyl-CoA-carboxylase	*accD*
	c-type cytochrome synthesis gene	*ccsA*
	Conserved open reading frames	*ycf1* ^b^ *, 2* ^b^ *, 3* ^a^ *, 4*

a Genes containing introns.

b Duplicated gene (genes present in the IR regions).

There are 16 genes with introns among the 112 genes of four *Polygonum* chloroplast genomes (Table 3), with 5 tRNA genes and 11 functional genes. The tRNA genes include *trnI-GAU*, *trnL-UAA*, *trnV-UAC*, *trnK-UUU*, *and trnA-UGG*. The 11 functional genes include *ndhB*, *ndhA*, *atpF*, *petB*, *petD*, *rpoC1*, *ycf3*, *clpP*, *rps12*, *rpl16*, and *rpl2*. The 5’ termination of the *rps12* gene is in the LSC region of the chloroplast genome, and the 3’ end is in the IR region of the chloroplast genome. Like the other angiospermous chloroplasts trans-splicing phenomenon also occurs in the *rps12* gene of the *Polygonum* chloroplast genome. Three of the 17 intron-containing genes cover two introns (*rps12*, *ycf3*, and *clpP*), and the other genes only contain one intron, of which *trnK-UUU* covers the main intron (2,510 bp), and this intron covers the entire gene of *matK*.

**TABLE 3:** Length of exons and introns in four *Polygonum* chloroplast genomes.

	Gene	Region	Exon 1	Intron 1	Exon 2	Intron 2	Exon 3
*Polygonum aviculare*	*atpF*	LSC	411	770	144	—	—
	*clpP*	IR	327	576	291	915	69
	*ndhA*	SSC	541	1099	551	—	—
	*ndhB*	IR	756	675	777	—	—
	*petB*	IR	6	769	642	—	—
	*petD*	IR	8	759	475	—	—
	*rpl16*	IR	399	931	9	—	—
	*rpl2*	IR	435	662	393	—	—
	*rpoC1*	LSC	1613	780	430	—	—
	*rps12*	IR	114	27587	232	527	26
	*trnA-UGC*	IR	38	801	35	—	—
	*trnI-GAU*	IR	42	945	35	—	—
	*trnK-UUU*	LSC	35	2510	37	—	—
	*trnL-UAA*	LSC	37	579	50	—	—
	*trnV-UAC*	LSC	37	583	38	—	—
	*ycf3*	LSC	155	744	227	739	128
*Polygonum bistorta*	*atpF*	LSC	411	770	144	—	—
	*clpP*	LSC	327	576	291	915	69
	*ndhA*	SSC	541	1099	551	—	—
	*ndhB*	IR	756	674	777	—	—
	*petB*	LSC	6	769	642	—	—
	*petD*	LSC	8	759	475	—	—
	*rpl16*	LSC	399	931	9	—	—
	*rpl2*	IR	435	662	393	—	—
	*rpoC1*	LSC	1613	780	430	—	—
	*rps12*	LSC+IR	114	75184	232	527	26
	*trnA-UGC*	IR	38	801	35	—	—
	*trnI-GAU*	IR	42	945	35	—	—
	*trnK-UUU*	IR	35	2510	37	—	—
	*trnL-UAA*	LSC	37	579	50	—	—
	*trnV-UAC*	LSC	37	583	38	—	—
	*ycf3*	IR	155	744	227	739	128
*Polygonum orientale*	*atpF*	LSC	411	770	144	—	—
	*clpP*	LSC	327	576	291	915	69
	*ndhA*	SSC	541	559	551	—	—
	*ndhB*	IR	777	675	756	—	—
	*petB*	LSC	6	796	642	—	—
	*petD*	LSC	8	759	475	—	—
	*rpl16*	LSC	399	931	9	—	—
	*rpl2*	LSC	1613	780	430	—	—
	*rpoC1*	LSC+IR	27	527	231	—	—
	*rps12*	LSC	114	75184	231	527	27
	*trnA-UGC*	IR	38	801	35	—	—
	*trnI-GAU*	IR	42	945	35	—	—
	*trnK-UUU*	LSC	35	2510	37	—	—
	*trnL-UAA*	LSC	37	579	50	—	—
	*trnV-UAC*	LSC	37	583	38	—	—
	*ycf3*	LSC	155	744	227	739	128
*Polygonum perfoliatum*	*atpF*	LSC	411	770	144	—	—
	*clpP*	LSC	327	576	291	915	69
	*ndhA*	SSC	541	1099	551	—	—
	*ndhB*	IR	756	675	777	—	—
	*petB*	LSC	6	769	642	—	—
	*petD*	LSC	8	759	475	—	—
	*rpl16*	LSC	399	931	9	—	—
	*rpl2*	IR	435	662	393	—	—
	*rpoC1*	LSC	1613	780	430	—	—
	*rps12*	IR	114	75080	232	527	26
	*rps16*	LSC	223	863	44	—	—
	*trnA-UGC*	IR	38	801	35	—	—
	*trnI-GAU*	IR	42	945	35	—	—
	*trnK-UUU*	LSC	35	2510	37	—	—
	*trnV-UAC*	LSC	37	583	38	—	—
	*ycf3*	LSC	155	744	227	739	128

### Relative Synonymous Codon Usage Analysis

Relative synonymous codon usage (RSCU) is a synonymous codon correlative effect, which values the 64 vital synonymous codons (Wu et al., 2007). RSCU is calculated as the ratio of the actual observed value to the average usage of the synonymous codons. The value of RSCU can be divided into three types: greater than 1, less than 1, and equal to 1. If the value of RSCU is greater than 1, it indicates that the codon is used more frequently than other codons. If the value of RSCU is less than 1, it means that other synonymous codons of this codon are used more frequently than this codon. If the value of RSCU is equal to 1, it indicates that there is no bias in the use of a codon. According to the statistical study of four *Polygonum* chloroplast genomes, the extent of CDS is from 80,286 to 83,403 bp, and for which accounts for about 50% of the total chloroplast genome length. The number of codons is in between 26,762–27,801. As for the statistical study of RSCU, there was some certain bias in the use of other amino acids, except for Trp and Met (Tables 4).

**TABLE 4:** Codon–anticodon recognition patterns and codon usage of four *Polygonum* chloroplast genomes

AA	Codon	RSCU value			
		*P. aviculare*	*P. bistorta*	*P. orientale*	*P. perfoliatum*
Stop	UAA	1.66	1.64	1.64	1.66
	UAG	0.76	0.73	0.75	0.67
	UGA	0.59	0.63	0.61	0.67
Ala	GCA	1.13	1.11	1.13	1.13
	GCC	0.7	0.74	0.72	0.68
	GCG	0.46	0.52	0.51	0.52
	GCU	1.7	1.64	1.64	1.68
Cys	UGC	0.52	0.54	0.59	0.55
	UGU	1.48	1.46	1.41	1.45
Asp	GAC	0.43	0.43	0.41	0.41
	GAU	1.57	1.57	1.59	1.59
Glu	GAA	1.5	1.46	1.46	1.46
	GAG	0.5	0.54	0.54	0.54
Phe	UUC	0.66	0.67	0.68	0.65
	UUU	1.34	1.33	1.32	1.35
Gly	GGA	1.54	1.56	1.53	1.57
	GGC	0.5	0.48	0.51	0.48
	GGG	0.74	0.74	0.73	0.7
	GGU	1.21	1.22	1.23	1.25
His	CAC	0.49	0.47	0.48	0.5
	CAU	1.51	1.53	1.52	1.5
Ile	AUA	0.93	0.97	0.96	0.95
	AUC	0.56	0.55	0.55	0.54
	AUU	1.51	1.48	1.49	1.51
Lys	AAA	1.49	1.48	1.48	1.49
	AAG	0.51	0.52	0.52	0.51
Leu	CUA	0.85	0.85	0.83	0.84
	CUC	0.43	0.45	0.42	0.42
	CUG	0.38	0.38	0.4	0.39
	CUU	1.29	1.26	1.31	1.28
	UUA	1.85	1.84	1.77	1.83
	UUG	1.21	1.22	1.27	1.24
Met	AUG	1	1	1	1
Asn	AAC	0.51	0.46	0.5	0.51
	AAU	1.49	1.54	1.5	1.49
Pro	CCA	1.04	1.12	1.07	1.04
	CCC	0.76	0.78	0.82	0.79
	CCG	0.69	0.61	0.6	0.62
	CCU	1.51	1.49	1.51	1.55
Gln	CAA	1.52	1.51	1.5	1.51
	CAG	0.48	0.49	0.5	0.49
Arg	AGA	1.68	1.76	1.69	1.71
	AGG	0.77	0.74	0.8	0.76
	CGA	1.43	1.42	1.43	1.4
	CGC	0.36	0.4	0.36	0.37
	CGG	0.47	0.42	0.47	0.48
	CGU	1.29	1.27	1.26	1.29
Ser	AGC	0.42	0.42	0.42	0.44
	AGU	1.17	1.2	1.16	1.16
	UCA	1.15	1.14	1.16	1.17
	UCC	0.97	1.03	0.99	0.97
	UCG	0.63	0.61	0.6	0.58
	UCU	1.66	1.6	1.66	1.67
Thr	ACA	1.23	1.23	1.27	1.25
	ACC	0.73	0.73	0.7	0.7
	ACG	0.51	0.55	0.52	0.53
	ACU	1.53	1.49	1.51	1.52
Val	GUA	1.46	1.43	1.44	1.46
	GUC	0.56	0.57	0.6	0.58
	GUG	0.54	0.55	0.54	0.53
	GUU	1.44	1.45	1.42	1.43
Trp	UGG	1	1	1	1
Tyr	UAC	0.38	0.39	0.41	0.41
	UAU	1.62	1.61	1.59	1.59

### Long-Repeat and Simple Sequence Repeat Analysis

**FIGURE 2:**
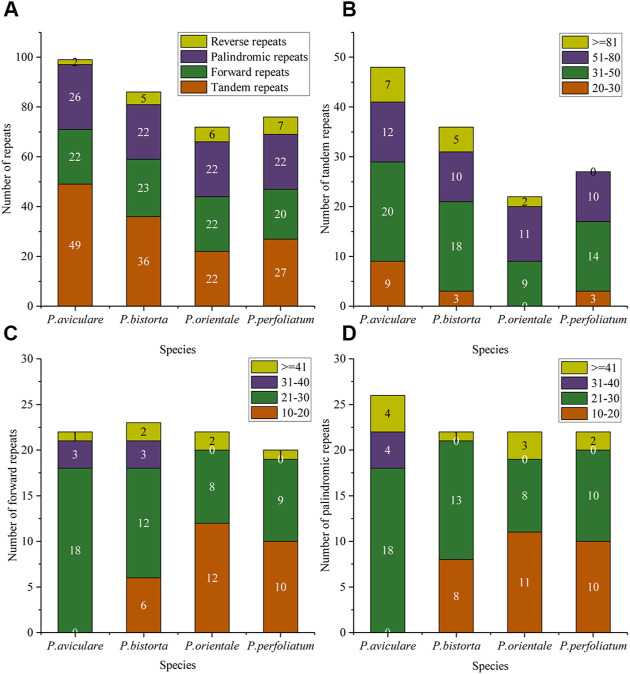
Repeat sequences analysis of the four *Polygonum* cp genomes. **(A)** Repeat types in the four cp genomes; **(B)** tandem repeats in the four cp genomes; **(C)** forward repeats in the four cp genomes; **(D)** palindromic repeats in the four cp genomes. In **(A)**, different colors show different repeat types; in **(B–D)**, different colors show different lengths. The ordinate represents the number of repeats.

In this research, we also studied the repeated sequence of four *Polygonum* chloroplast genomes, with tandem repeats (T), forward repeats (F), reverse repeats (R), and palindromic repeats (P). The results of the repeated study of four *Polygonum* chloroplast genomes are in Figure 2. There are 99 repeated sequences in *P. aviculare*, including 49 tandem repeats, 22 forward repeats, 26 palindromic repeats, and 2 reverse repeats; 86 repeated sequences in *P. bistorta*, including 36 tandem repeats, 23 forward repeats, 22 palindromic repeats, and 5 reverse repeats; 72 repeated sequences in *P. orientale*, including 22 tandem repeats, 22 forward repeats, 22 palindromic repeats, and 6 reverse repeats; 76 repeated sequences in *P. perfoliatum*, including 27 tandem repeats, 20 forward repeats, 22 palindromic repeats, and 7 reverse repeats. Among all the categories of repeated sequences, the sequences of length 20–50 bp are the most (Figure 2).

**FIGURE 3:**
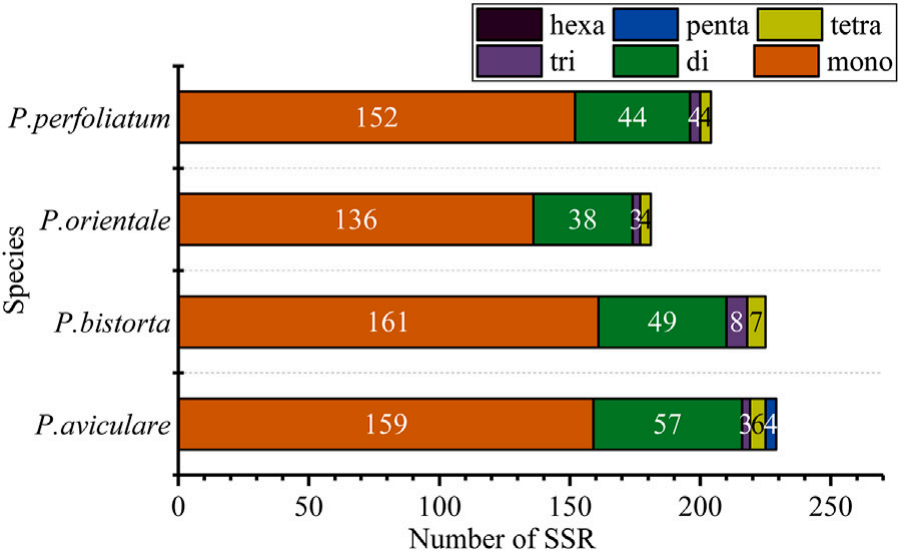
SSR analysis of four cp genomes. The ordinate represents the number of SSRs.

Simple sequence repeat (SSR), also known as microsatellite sequence, is a repeat sequence composed of one to six bases as repeat units in series, which is of great significance to the study of plant populations. SSRs with a length of over 10 bp are inclined on slipped-strand mispairing, which is approved to be the principal mutational mechanism of SSR polymorphisms (Asaf et al., 2017). Additionally, SSRs which are changeable at the intraspecific position in the chloroplast genome are often used as the genetic marker in the investigation of population genetics and evolution (Yang et al., 2016; Zl et al., 2016). There are 228 SSRs in *P. aviculare*, including, three trinucleotides, 57 dinucleotides, 159 mononucleotides, 6 tetranucleotides, and 3 pentanucleotides; 225 repeated sequences in *P. bistorta*, including 7 tetranucleotides, 8 trinucleotides, 161 mononucleotides and 49 dinucleotides; 181 repeated sequences in *P. orientale*, including 136 mononucleotides, 38 dinucleotides, 3 trinucleotides and 4 tetranucleotides; 204 repeated sequences in *P. perfoliatum*, including 152 mononucleotides, 44 dinucleotides, 4 trinucleotides, and 4 tetranucleotides (Figure 3). Based on these SSR results, we designed 10 pairs of primers as molecular markers. *P. orientale*, *P. cuspidatum*, and *P. aviculare* were used for molecular marker amplification. The results showed that four pairs of primers could distinguish well between species (Supplementary Figure S1). Although the four of them can be distinguished at the genus level, the PCR amplification products may be chloroplast DNA or nuclear DNA, which requires further study.

### Comparative Chloroplast Genomic Analysis and Sequence Variation

**FIGURE 4:**
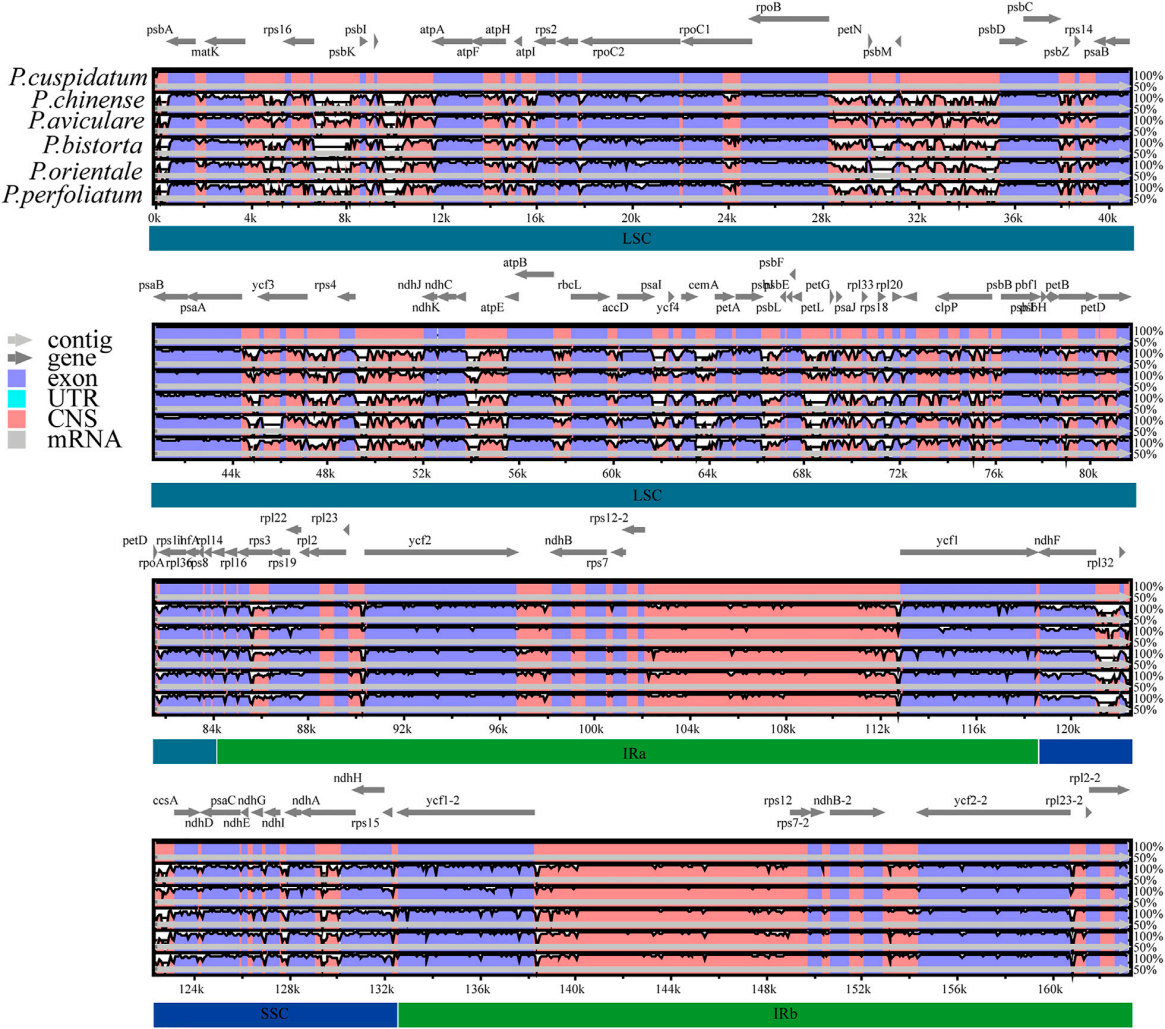
Comparative analysis of chloroplast genome differences in six *Polygonum* cp genomes. Gray arrows and thick black lines above the alignment indicate gene orientation. Purple bars represent exons, blue bars represent untranslated regions (UTRs), pink bars represent non-coding sequences (CNS), and gray bars represent mRNA. The y-axis represents the percentage identity (shown: 50–100%).

**FIGURE 5:**
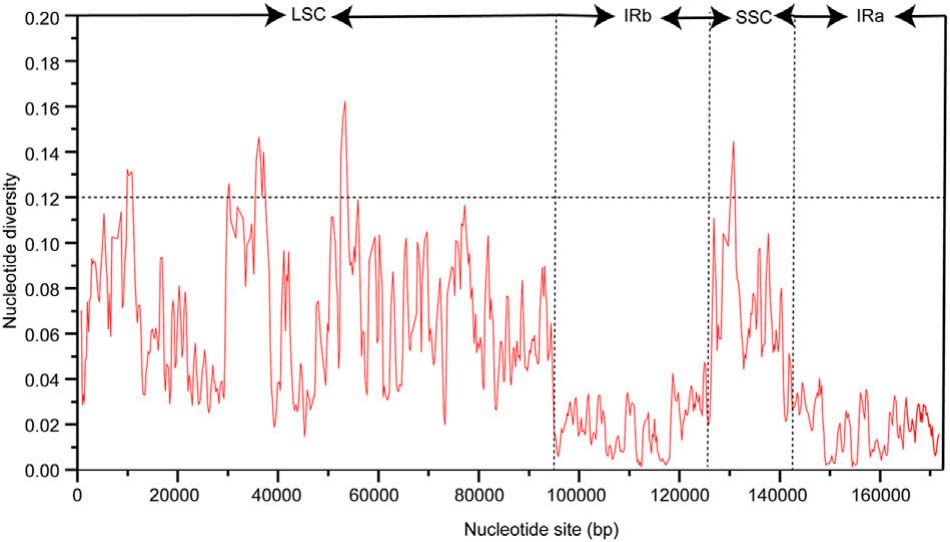
Nucleotide diversity (Pi) among cp genomes of six *Polygonum* species.

The chloroplast genome sequence of *P. cuspidatum* is used as *a* reference sequence to draw an analogy among the genomic sequences of six *Polygonum* chloroplast genomes (*P. cupidatum, P. chinense, P. aviculare, P. bistorta, P. orientale,* and *P. orientale*). Figure 4 displayed regions in common. The figure displayed that, aiming at the *Polygonum* chloroplast genomes; the rate of changes for the LSC and SSC regions is visibly greater than that of the IR region. To further clarify the variation in the coding regions, the Pi (nucleotide diversity) was also calculated (Figure 5). Six divergent loci (*psbI-trnS-GCU*, *rpoB-trnC-GCA*, *trnE-UUC-trnT-GGU*, *trnT-GGU-psbD*, *trnT-UGU-trnL-UAA*, and *rpl32-trnL-UAG*) had a Pi value greater than 0.12. All of these six divergent loci were intergenic regions and were present in the LSC region, except for *rpl32-trnL-UAG*, which occurred in the SSC region, with none being detected in the IR region. These highly variable regions may also resolve the interspecific relationships of *Polygonum* in the Polygonaceae phylogeny. With the forward progress of the chloroplast genome because of the wide usage of the chloroplast DNA fragments, some conundrums in plant species authentication, phylogenetics, and other related researche studies can be solved. Obviously, the chloroplast DNA fragments have higher resolution loci than general DNA fragments which have low distinguishability and mutation rates in some proximal related groups. Meanwhile, these high-efficiency DNA fragments from the chloroplast genome can promote the development of species identification and population diversity by comparing the differences between the chloroplast genome sequences of different groups.

IR Contraction and Expansion in *Polygonum* Chloroplast Genome

**FIGURE 6:**
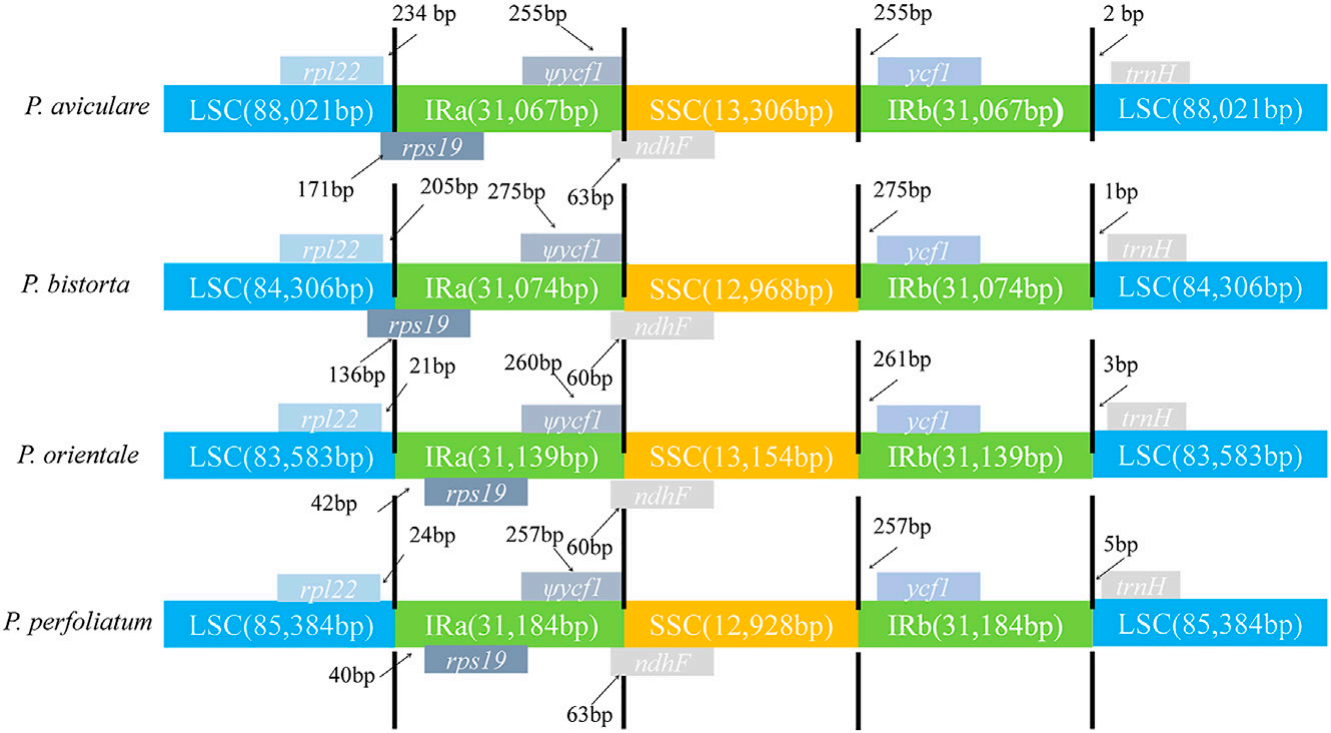
Comparison of the chloroplast genome boundaries in four *Polygonum* cp genomes.

In this research, the analysis of IR-LSC and IR-SSC border structure and location of four *Polygonum* species was finished (Figure 6). The results found out that the SSC/IRa assembly was located in the *ndhF* region in the four species of *Polygonum* chloroplast genome, and spread a length of 60–63 bp into the IRa region in the four species. Also, the *rps19* gene was located in the IRa region and the length is about (*P. orientale*, 21 bp; *P. perfoliatum*, 40 bp) into the LSC region. The *ycf1* gene of four *Polygonum* chloroplast genomes completely exists in the IRb region, with a terminal 225 bp (*P. aviculare*), 275 bp (*P. bistorta*), 261 bp (*P. orientale*), and 257 bp (*P. perfoliatum*) from the SSC/IRa border. For now, the *trnH* gene existed in the LSC region, and it had a length of 2, 1, 3, and 5 bp from the LSC/IRb border in the chloroplast genome of *P. aviculare, P. bistorta*, *P. orientale*, and *P. perfoliatum*, individually.

Until now, the expansion mechanism of the IR region has been debated; moreover, the double-strand break repair (DCBR) theory is written off as the prime mechanism for the expansion of the IR region (Machour and Ayoub 2020). There is little probability of the IR region, which has a large shrink. Furthermore, it is believed that the DCBR model is not only the core mechanism of IR region expansion but also the mechanism of IR region contraction.

### Phylogenetic Analysis

**FIGURE 7:**
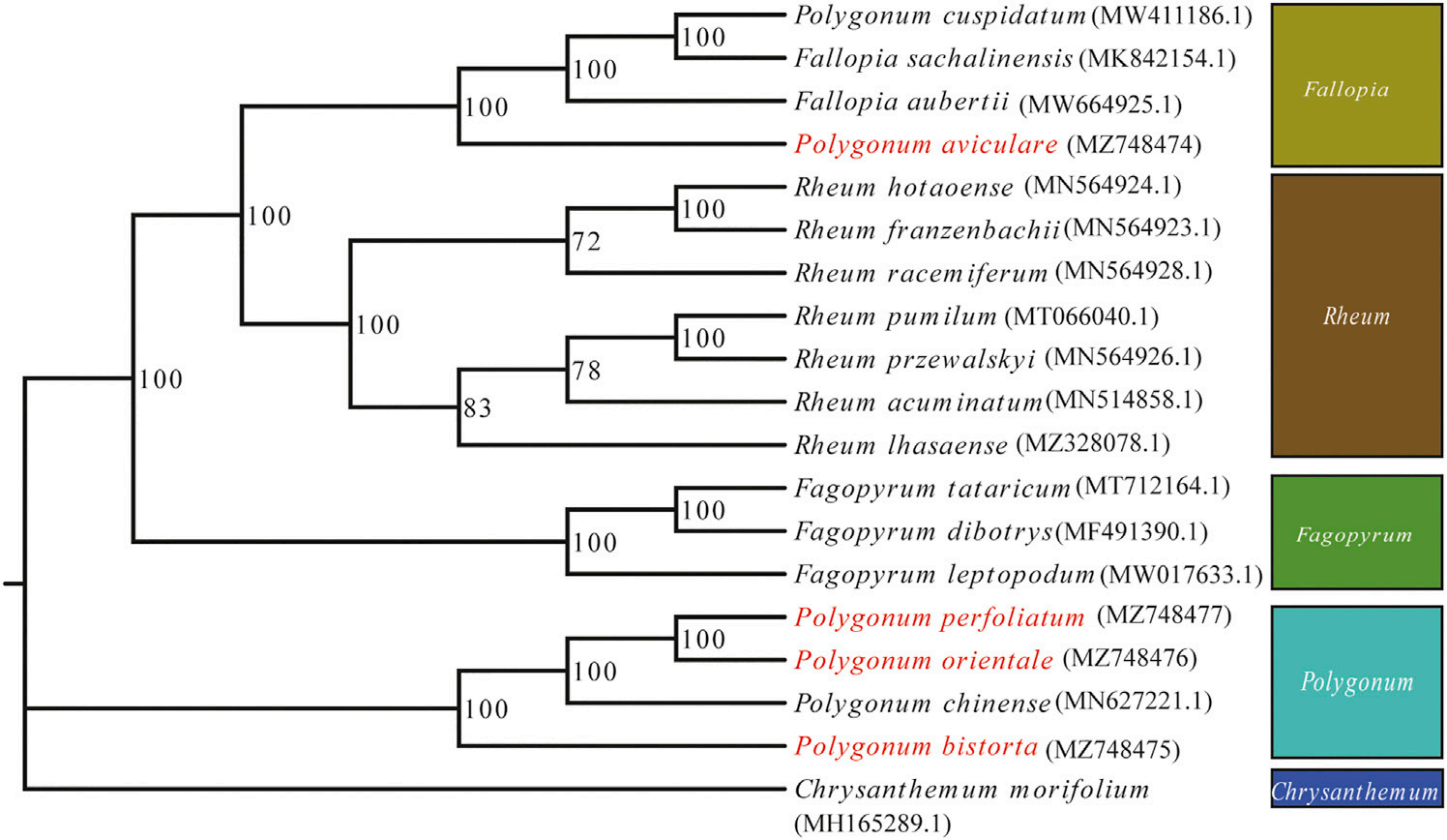
ML phylogenetic tree reconstruction containing the cp genomes of 19 plants. *Chrysanthemum x morifolium* was set as the out-group.

Phylogenetic analysis was accomplished on an alliance of concatenated nucleotide sequences of all genes from 19 angiosperm species. We used ML to construct a phylogenetic tree ground based on these gene data, while *C. morifolium* was agreed as the outgroup. The *P. bistorta*, *P. orientale*, *P. perfoliatum*, and *P. chinense* are divided into a branch, namely, *Polygonum*. And in the branch of *Polygonum*, it is further divided into two small sub-branches. The upper sub-branch includes *P. orientale*, *P. perfoliatum*, and *P. chinense*. Moreover, *P. orientale* and *P. perfoliatum* clustered on the small branch. The *P. aviculare* is closer to *F. sachalinensis*, *F. aubertii*, and *Polygonum cuspidatum*, which is far away from the branch of *Polygonum*. Therefore, we put *P. aviculare* and *P. cuspidatum* into the branch of *Fallopia* (Figure 7).

## Discussion

Overall, we examined the four species of *Polygonum* chloroplast genomes, and then the results inferred that the four *Polygonum* species were similar in the angiospermous features both in structure and content. Also this shows that the characteristics of the chloroplast genome in other medicinal angiosperms (He et al., 2017) would be reliable with the characteristic quadripartite structure of the *Polygonum* chloroplast genome. The phenomenon is general among other angiospermous chloroplast genomes (Raubeson et al., 2007; Yang et al., 2010; Asaf, Khan, Khan, Waqas, Kang, Yun and Lee 2017) that the AT content was higher than the GC content in the chloroplast genome among all four *Polygonum* species, then entirely these presented that there were no significant variances in chloroplast genomes among those four *Polygonum* species. The consequences also confirmed that the GC content was much higher, probably because of the presence of the huge quantity of rRNA in the IR regions. However, the precise details are still poorly unknown. The consequences turned up in coding and extremely different regions among *Polygonum* chloroplast genomes were also exposed among other floristic chloroplast genomes (Ni et al., 2016; Chen et al., 2017).

The length of introns and exons was important among various plant chloroplast genomes. Here, the results indicated that only one gene (*rps12*) included three exons, meanwhile two genes (*ycf3* and *clpP*) had two introns among the four *Polygonum* chloroplast genomes. The *rps12* genes’ were located at the 5’ end on the LSC region and meanwhile the duplicated 3’ ends was set in the IRs regions due to the phenomenon it has been called trans-spliced gene (Guo, Guo, Zhao, Xu, Li, Zhang, Shen, Wu and Hou 2018). Furthermore, *ycf3* is known as a photosynthesis-related gene as reported before (Naver et al., 2001). Therefore, the attendance of the *ycf3* gene might result in an extra study of *Polygonum* chloroplast. The *ycf1* gene also expected a basic part in the chloroplast genome, there were some related studies focused on gene capacity around ycf1, and these reports exposed ycf1 as a paramount pseudogene for those varieties of chloroplast genome and similarly encodes for Tic214 in plants (de Cambiaire et al., 2006; Nakai 2015). It has been testified in an earlier study that the introns play a significant part in regulating the expression of genes (Xu et al., 2017), which might control the gene expression level in different spatiotemporal (Le Hir et al., 2003; Niu and Yang 2011). Additionally, we reached a status that the attendance of intron or the loss of genes can be discovered in the chloroplast genomes (Graveley 2001; Ueda et al., 2007), and the regulating function of intron similarly need to be exposed through the research among a large number floristic chloroplast genomes (Emami et al., 2013). However, currently, there was no related research on the intron regulation mechanisms among those *Polygonums*. So, we will obtain more appropriate information through further studies to find out the functions of introns in the chloroplast genomes. And, these data around chloroplast genome will entitle significant theoretical basis for plant identification, especially medicinal plants.

It is important to identify the resources of plant germplasm and molecular markers by the finding the long repeat sequences and the SSRs of the chloroplast genomes. The results of the study demonstrated that the genes which have long repeats may be produced as a genetic marker for identifying the related species, but the exact use of it still needs to be proved by further studies. SSRs play a crucial status role in the chloroplast genomes. Because of its extreme variability, it was always used in genetic research (Ebert and Peakall 2009; Dong et al., 2013). The previous studies presented that SSR is widely distributed in the genome, and because of its special parental inheritance features, SSR is usually analyzed for genetic population structure and maternal analysis. Former researchers have studied that *A. formosae* has the most plentiful repeats on mononucleotides, and the phenomenon among the four *Polygonum* chloroplast genomes was in common. Consequently, the development found in SSRs of chloroplast genomes will significantly inspire the learning of identification among plenty species, genetic variety, and evolutionary development in *Polygonum* (Provan 2000; Flannery et al., 2006).

The method of DNA barcoding, which was put forward by Hebert (Hebert et al., 2003; Hu et al., 2019), can be utilized to identify the species through DNA sequences, *ITS2, matK, psbA-trnH,* and *rbcL*. However, the identification of related species—and predominantly the morphologically confusing species in the same genus—still exist in various problems. For that reason, discovering a proper DNA marker for such species is indispensable. The cp genomes have habitually been utilized for phylogenetic studies and species identification as a result of they have slower evolution than nuclear genomes (Song et al., 2017). In the current study, an analysis of five *Polygonum* cp genomic alignments has shown an enlarged figure of mutable sites in the intergenic spacer of the *atpI-rps2*, *atpB-rbcL*, *psbD-rps14*, *ycf4-cemA*, etc. Thus, these regions may be utilized as different nominee fragments to identify *Polygonum*. Moreover, *ycf1a* or *ycf1b* is the most mutable plastid genome region and can be used as a principal barcode for land plants (Dong et al., 2015). On the other hand, more *Polygonum* cp sequence data is necessary to be tested and it should be handled in future studies.

Earlier research studies had shown that IRs regions were the most conserved regions in the chloroplast genomes (Daniell et al., 2016). Its shrinkage and expansion at the borders are a common evolutionary occasion, and characterize the governing cause for the size variation and rearrangement of the chloroplast genome. There were a lot of reports that showed that the chloroplast gene had been conserved in most land plants but there were also reports that many sequences were rearranged in the chloroplast genomes of most plant species, then the IR shrinkage and extensions with inversions, the inversions in the LSC region, and the re-inversion in the SSC region were involved, and some reports showed that the wide rearrangements in the chloroplast genome of *Trachelium caeruleum* are associated with repeats and tRNA genes (Raubeson and Jansen 2005; Haberle et al., 2008). The sequence rearrangements caused by the alteration of chloroplast genome structure in related species may be connected with the plant genetic multiplicity information, so it can be utilized for molecular identification and evolutionary study (Chumley et al., 2006).

At present, it has been known that the chloroplast genome can serve as a super barcode to identify the plant species (Hernández-León et al., 2013). By phylogenetic analysis of the chloroplast genome of four *Polygonum* species, we suggested that the chloroplast genome of *Polygonum* might be a key marker for species identification. Furthermore, research is necessary to study and identify this assumption. The results are of great value to the genetic diversity and phylogenetic research of *Polygonum* at some point. Nevertheless, our research did not completely figure out the relationship between genera. Furthermore, our phylogenetic study is grounded on the chloroplast genome. If we want to completely figure out the phylogeny of the species in *Polygonum*, we may need to study the nuclear genes of plants, and more genera should be involved in the future. Nonetheless, our phylogeny research provided treasured resources for the classification, phylogeny, and evolutionary history of *Polygonum*.

## Data Availability

The datasets presented in this study can be found in online repositories. The names of the repository/repositories and accession number(s) can be found at: https://www.ncbi.nlm.nih.gov/genbank/, MZ748474 https://www.ncbi.nlm.nih.gov/genbank/, MZ748475 https://www.ncbi.nlm.nih.gov/genbank/, MZ748476 https://www.ncbi.nlm.nih.gov/genbank/, MZ748477 https://www.ncbi.nlm.nih.gov/, SRR15604831 https://www.ncbi.nlm.nih.gov/, SRR15604830 https://www.ncbi.nlm.nih.gov/, SRR15604829 https://www.ncbi.nlm.nih.gov/, and SRR15604828.
