# Supplementary material for: A Comparative Analysis of the Chloroplast Genomes of Four Polygonum Medicinal Plants
Source: Front Genet. 2022 Apr 25;13:764534. doi: 10.3389/fgene.2022.764534 (PMC9084321; doi:10.3389/fgene.2022.764534)
Supplement: Supplementary file 1 [file DataSheet1.DOCX]

**Supplementary Table 1. Information on four samples of *Polygonum.***

| **Sample** | **Organ** | **Species** | **Locality** | **Collection Date** | **Voucher** |
| --- | --- | --- | --- | --- | --- |
| HBGP0684 | Leafs | *Polygonum aviculare* | Sichuan, China | 20201005 | gs2018100503 |
| HBGP0685 | Leafs | *Polygonum* *bistorta* | Sichuan, China | 20201020 | gs2018102012 |
| HBGP0688 | Leafs | *Polygonum* *orientale* | Sichuan, China | 20201113 | gs2018111304 |
| HBGP0689 | Leafs | *Polygonum orientale* | Sichuan, China | 20201128 | gs2020112811 |

**Supplementary Table 2. DNA sequencing data generation summary of *Polygonum.***

| **Sample** | **Platform** | **Reads number** | **Data collected (Gb)** | **Reads length(bp)** |
| --- | --- | --- | --- | --- |
| HBGP0684 | Illumina | 11991533 | 3.5 | 300 |
| HBGP0685 | Illumina | 12712837 | 3.8 | 300 |
| HBGP0688 | Illumina | 11687951 | 3.5 | 300 |
| HBGP0689 | Illumina | 19116150 | 5.7 | 300 |

**Supplementary Table 3. Primers used for assembly validation.**

| **Target area** | **Primer** | **Primer sequence (5′-3′)** | **Tm** | **Annealing temperature** |
| --- | --- | --- | --- | --- |
| trnS-rps4 | trnS(F) | AGGCGTAGCATTGGAACT | 52.7 | 50 |
|  | rps4(R) | CATATAAAGGAGTAGTCAATCA | 47.1 |  |
| trnD-trnY | trnD(F) | ACCAATTGAACTACAATCCCA | 54.6 | 52 |
|  | trnY(R) | CTACGCTGGTTCAAATCC | 50.7 |  |
| rpoB-trnC | rpoB(F) | AAAACTTGAGATAATGGATGTAA | 51.4 | 56 |
|  | trnC(R) | AAGGATTTGCAGTCCTCCGCCT | 66.2 |  |
| trnE-trnT | trnE(F) | CTCCTTGAAAGAGAGATGTCCTGAA | 61.2 | 53 |
|  | trnT(R) | ATGGCGTTACTCTACCACTGA | 54.9 |  |
| trnP-psaJ | trnP(F) | AAACGCGCTACCAAGCTGCG | 66.0 | 56 |
|  | psaJ(R) | GAGATATGTTTTTAGATCTCGC | 51.1 |  |

**Supplementary Table 4. 19 angiosperm species and the chloroplast genomes information.**

| **Species** | **Accession** | **Species** | **Accession** |
| --- | --- | --- | --- |
| *Chrysanthemum morifolium* | MH165289.1 | *Polygonum Cuspidatum* | MW411186.1 |
| *Fagopyrum dibotrys* | MF491390.1 | *Polygonum perfoliatum* | MZ748477 |
| *Fagopyrum leptopodum* | MW017633.1 | *Rheum acuminatum* | MN514858.1 |
| *Fagopyrum tataricum* | MT712164.1 | *Rheum franzenbachii* | MN564923.1 |
| *Fallopia aubertii* | MW664925.1 | *Rheum hotaoense* | MN564924.1 |
| *Fallopia sachalinensis* | MK842154.1 | *Rheum lhasaense* | MZ328078.1 |
| *Ploygonum orientale* | MZ748476 | *Rheum przewalskyi* | MN564926.1 |
| *Polygonum aviculare* | MZ748474 | *Rheum pumilum* | MT066040.1 |
| *Polygonum bistorta* | MZ748475 | *Rheum racemiferum* | MN564928.1 |
| *Polygonum chinense* | MN627221.1 |  |  |

**
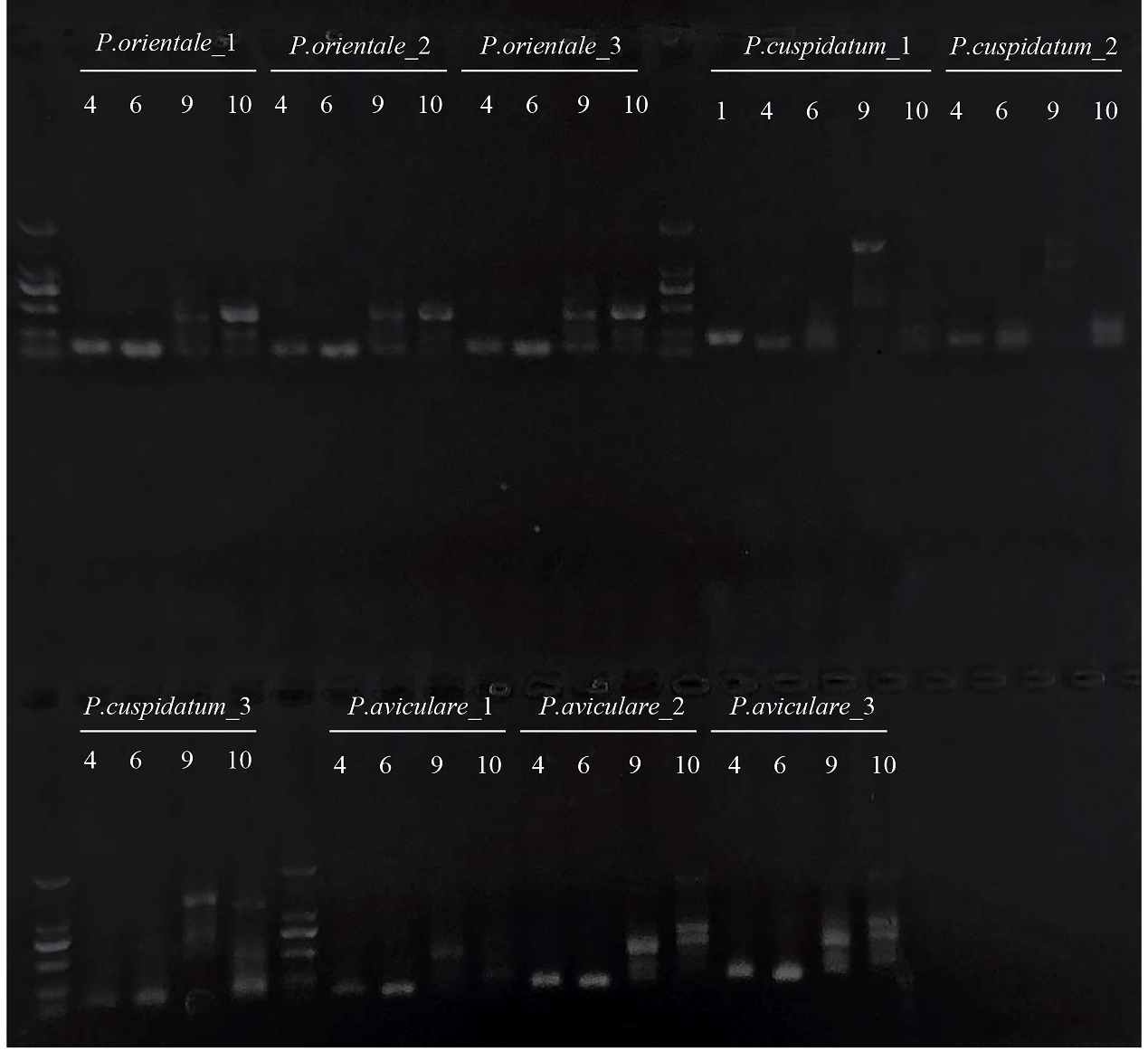
**

**Supplementary Figure 1. SSR molecular marker gel imaging results.**
